# Characterization of a Novel High Internal Phase Pickering Emulsions Stabilized by Soy Protein Self-Assembled Gel Particles

**DOI:** 10.3389/fnut.2021.795396

**Published:** 2021-12-23

**Authors:** Chong-hao Bi, Shang-yi Chi, Tong Zhou, Xue-ying Wang, Jia-yi Zhang, Zhi-gang Huang, Fei Gao

**Affiliations:** ^1^School of Artificial Intelligence, Beijing Technology and Business University, Beijing, China; ^2^Beijing Key Laboratory of Quality Evaluation Technology for Hygiene and Safety of Plastics, Beijing, China; ^3^School of Food and Health, Beijing Technology and Business University, Beijing, China

**Keywords:** acid-induced self-assembled gels, emulsion gels, rheological properties, thermal properties, microstructure

## Abstract

In this paper, a novel high-internal-phase Pickering emulsion (HIPPE) prepared by acid-induced self-assembly SPI gel (A/S-SPIG) was investigated. The steady-state shear test results showed that all HIPPEs were typical shear thinning emulsion, which could form stable emulsion (0.2–1.2% SPI concentration). The network structure of HIPPE stabilized by A/S-SPIG particles (0.2–1.2% SPI concentration) was continuously enhanced with increasing SPI concentration. The high concentration of SPI particles increased the crystallization temperature of the stabilized HIPPE. Meanwhile, at a concentration of 1.2%, HIPPE has the best cohesive property and stability against delamination due to weakened mobility. In conclusion, A/S-SPIG was proved excellent HIPPE stabilized particle.

## Introduction

Pickering emulsions, as solid particle stabilized emulsions, show to have long-term stability and many unique advantages over ordinary emulsions (1–3). The high internal phase emulsion stabilized by solid particles is also called “high internal phase Pickering emulsion” (HIPPE), and the oil concentration is generally >74% (4). The advantages of HIPPE include less amount of stabilizer required, high stability against coalescence, high storage stability, and less environmental pollution (5, 6). Protein-based particles have a promising future as HIPPE stabilizers because they do not require additional surface modification and are suitable for high-pressure homogenization (7).

Self-assembly of proteins is the formation of various nanostructures with other components by non-covalent interactions such as hydrogen bonds, electrostatic interactions, hydrophobic interactions and van der Waals interactions, as well as metal ions (8, 9). Soybean proteins isolated (SPI) can form an ordered structure to complete the self-assembly process by lowering the pH value of the environment (10, 11) investigated the effects of pH and concentration on the self-assembly of soy globulin in aqueous solution and showed that the size of the self-assembled aggregates increased with decreasing pH and increasing concentration. Liu and Tang (12) formed nanoparticle aggregates by heating and electrostatic screening of soybean isolates, and emulsions stabilized by these nanoparticle aggregates have strong emulsification stability. Tian et al. (13) reported that the emulsification efficiency of β-conglycinin (7S) was positively correlated with concentration, and showed better emulsification efficiency at pH values of 3 and 8. Xu et al. (14) reported that natural soybean β-conglycinin can be used as outstanding Pickering stabilizers for oil-in-water HIPPE. Increasing protein concentration can increase the stiffness of the HIPPE. The formation of HIPPE has excellent temperature responsiveness and can remain stable during long-term storage of 60 days. Liu et al. (15) developed HIPPE with 75% oil content using bacterial cellulose nanofiber/isolated soy protein (BCNs/SPI) composite colloidal particles. Compared with SPI, the rheology, crystallinity, thermal stability, and wettability of BCN/SPI colloidal particles were improved. By increasing the concentration of BCNs/SPI composite colloidal particles, the stability of their stabilized HIPPE can be improved. Peng et al. (16) reported a novel antioxidant HIPPE (oil content ϕ > 0.74) stabilized with soybean β-conglycinin (β-CG) and polyphenol composite nanoparticles. These HIPPE are very stable after heating or long-term storage, and the prepared HIPPE shows an excellent protective effect on β-carotene (encapsulated in the oil phase) to prevent heating and inhibit lipid oxidation.

To date, most protein particles used for the preparation of HIPPEs require additional particles or substances as stabilization aids, and examples of HIPPEs stabilized by only a single type of particle have rarely been studied (17). In previous studies, we found that acid-induced self-assembled SPI gel (A/S-SPIG) have potential as Pickering particles (18). In this study, the A/S-SPIG was broken into particles by high-speed shearing and used as high internal phase Pickering emulsion stabilizer. The rheology, freeze-thaw stability, crystallinity, particle size and microstructure of HIPPE stabilized by A/S-SPIG were then tested. We finally proposed a novel manufacturing strategy for food-grade protein particle-stabilized HIPPE.

## Materials and Methods

### Materials

Soy protein isolate (dispersed, SPI > 90 %) was purchased from Shanghai Macklin Biochemical Co., Ltd, the glucono-δ-lactone (GDL, BR) was bought from Shanghai Macklin Biochemical Co., Ltd, and soybean oil was obtained from Jinlongyu Food Co., Ltd, Nile Red Reagent was bought from Shanghai McLean Biochemistry Co., Ltd.

### Experimental Methods

#### Preparation of A/S-SPIG

SPI was prepared using a magnetic stirrer (IKA Instruments, Germany) by dissolving SPI in deionized water (pH = 7.0 ± 0.3) at 25°C for 0.5 h. The SPI stock solution containing 6% (w/w) concentration was heated to 90°C in a water bath with a magnetic stirrer to completely denature the protein to make SPI dispersion. After that, GDL (1.5% w/w) was added to the SPI dispersion and heated in a water bath at 50°C for 20 min to form A/S-SPIG (19). Then the A/S-SPIG was crushed into micron-sized A/S-SPIG particles by a high-speed shear (German IKA Instruments), which (0.2–1.2% w/w) was added to oil-water mixtures with different oil phase ratios (ϕ = 0.1–0.9), and finally sheared at 12,000 rpm for 2 min (20).

#### Appearance Observation and Fat Floating Index (CI%)

The stability characteristics of the HIPPE is characterized by the appearance photograph and the Creaming Index (CI%) (21). After the emulsion is prepared, immediately take 10 mL in a flat-bottomed test tube (1.5 ×12 cm), record the layering of the emulsion under room temperature storage (height Hs and total height Ht of the subnatant) and take a picture. The CI% values is calculated as follows:


(2 – 1) $$\begin{array}{l} \text{CI}\%=\left(\text{Hs/Ht}\times 100\%\right). \end{array}$$

#### Rheological Testing

##### Steady Shear Tests

The shear viscosity of the HIPPE with different protein concentrations was characterized by a TA rheometer (DHR-2) equipped with parallel plates. The diameter of the plate *d* = 40 mm, the distance between the plates was set to 1 mm, and the test temperature was 25°C. Take a sample of about 1 mL and place it between the two plates, wipe off the excess sample outside the plate, and record the viscosity change with the shear rate in the range of 1–200 s^−1^ through the TRIOS software.

##### Frequency Scanning Tests

Viscoelasticity of different protein stabilized HIPPE using the same rheometer using frequency scanning mode. The frequency scan range is set at 0.5–500 rad/s, the strain is set to 0.5% (the set value is in the LVR) to ensure that all measurements are in the linear viscoelastic region. The measurement results were recorded by TRIOS software and expressed as storage modulus (*G'*) and loss modulus (*G”*), respectively.

The storage modulus (*G'*) and loss modulus (*G”*) of A/S-SPIG stability emulsion can be fitted by power law function:


(2 – 2) $$\begin{array}{l} \;G^{{\;}^{\prime }}=k^{{\;}^{\prime }}\cdot w^{n^{{\;}^{\prime }}} \end{array}$$

(2 – 3) $$\begin{array}{l} G^{{\;}^{\prime \prime }}=k^{{\;}^{\prime \prime }}\cdot w^{n^{{\;}^{\prime \prime }}} \end{array}$$

Where *K* is the power law constant (Pa.sn), n is the frequency index, and ω is the angular frequency (rad/s) (19).

##### Creep Recovery Test

The creep recovery test was conducted at a temperature of 25°C. First, a constant torque of 100 μN·m was applied, and the changes in the compliance and strain of the stable HIPPE with different protein concentrations were recorded within 2 min. The stress was released after 2 min, and the changes in the compliance and strain of the stable Pickering emulsion within 2 min were recorded.

The creep compliance curve of A/S-SPIG stabilization Pickering emulsion can be fitted with a four-element Maxwell-Voigt model:


(2 – 4) $$\begin{array}{l} J\left(t\right)=\frac{1}{G_{H}}+\frac{1}{G_{V}}\left(1-e^{-\frac{t}{\tau }}\right)+\frac{t}{\eta_{N}} \end{array}$$

(2 – 5) $$\begin{array}{l} \tau =\frac{\eta_{V}}{G_{V}} \end{array}$$

In the formula, *J* represents compliance (1/Pa), which is equal to strain divided by stress; *G*_*H*_ and *G*_*V*_ represent Hook element's elastic modulus (Pa) and Voigt element's elastic modulus (Pa), respectively; τ is hysteresis time (S); η_*V*_ and η_*N*_ represent the viscous part (Pa·s) in the Voigt element and the viscous part (Pa·s) in the Newtonian element, respectively (22).

The recovery rate of the HIPPE was calculated based on the creep curve of the A/S-SPIG stable Pickering emulsion. Recovery rate R(%) can be calculated using the following formula:


(2 – 6) $$\begin{array}{l} R\left(\text{\%}\right)=\frac{\gamma_{\tau }\text{-}\gamma_{\text{p}}}{\gamma_{\tau }}\times 100 \end{array}$$

Where γτ (%) and γ*p* (%) represent total strain and recovery strain, respectively (23).

#### Particle Size Distribution of HIPPE Droplets

Measure the droplet size of freshly prepared emulsion samples, using Mastersizer 3000 particle size distribution analyzer (Shanghai Subway Instrument System Co., Ltd). Dispersant is deionized water, relative refractive index of emulsion is 1.470, refractive index of dispersant is 1.330, speed of agitator is 2,450 rpm. During the measurement, it is necessary to fully stir the diluted emulsion to make it evenly distributed to avoid a large number of bubbles or undispersed emulsion droplets in the dispersant. For highly flocculated or coalesced emulsions, you must be careful to choose a suitable stirring paddle speed to ensure the integrity of the emulsion droplet flocculation structure. The final measurement result is expressed by the volume average droplet size *d*_4,3_ and the surface area average size *d*_3,2_. Each measurement of the emulsion sample is repeated three times to take the average value. The calculation formula is as follows:


(2 – 7) $$\begin{array}{l} d_{4,3}=\sum n_{i}d_{i}^{4}\sum n_{i}/d_{i}^{3} \end{array}$$

(2 – 8) $$\begin{array}{l} d_{3,2}=\sum n_{i}d_{i}^{3}\sum n_{i}/d_{i}^{2} \end{array}$$

#### Thermal Stability Test of HIPPE

DSC thermal analysis of freshly prepared emulsions (Differential Scanning Calorimetry, TA Instruments). Each emulsion sample was removed with a pipette immediately after preparation. Five to ten milligram sample were weighed to a TA Q20 liquid aluminum crucible with an electronic analytical balance. Then, the liquid aluminum crucible was covered by a matched aluminum cover pressing plate. A sample plate is placed on the electrode outside the DSC chamber. a sealed aluminum plate without sample was placed on the inside for control. Two aluminum crucibles were placed in the DSC furnace cavity, cooled from 40 to −40°C and then heated to 40°C, the heating and cooling rates were both 10°C/min, and the nitrogen flow rate was 40 mL/min.

#### Microstructure of HIPPE

The microscopic observation was used confocal laser scanning microscope (LEICA Instruments Germany, TCS-SP8). In the confocal laser scanning microscope test, the excitation wavelength of Nile Red is 530 mm. The objective lens used 40 × 0.85 HCPL APO lens.

#### Data Analysis

After the experiment, SPSS software was used for data processing, single factor analysis and Duncan test were used to analyze the significant differences. At least three parallel repeated experiments are done for each emulsion sample.

## Result and Discussion

### HIPPE Delamination and Fat Floating

**Figure 1:**
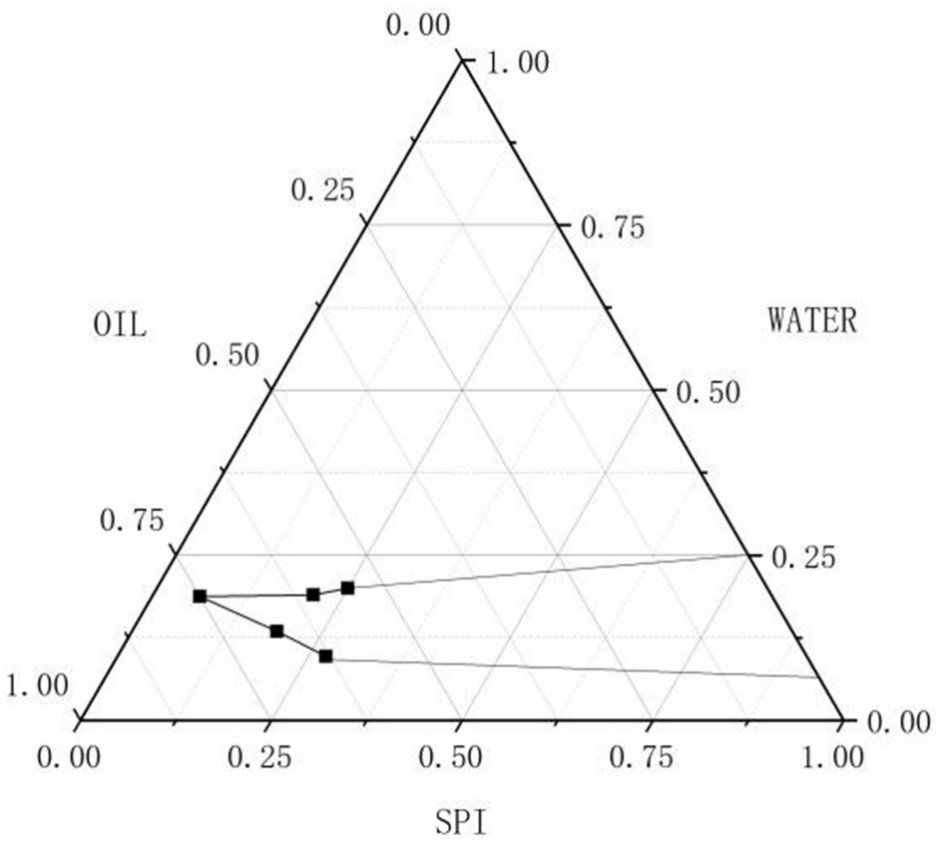
Three-phase diagram of stable Pickering emulsion under different SPPI particle concentration.

Pickering emulsion is a three-phase mixed system. In the ternary phase diagram, the oil phase, the aqueous phase, and the A/S-SPIG particles are the three vertices of the phase diagram. Combined with the enclosed area of the figure, the formation of Pickering emulsion can be clearly distinguished. As shown in the Figure 1, the mixed system can form fine Pickering emulsions at high oil ratios. At an oil ratio of 80%, less A/S-SPIG particles are needed to form a stable and high-quality Pickering emulsion. This ratio played a key role in screening A/S-SPIG particles as a suitable formulation for Pickering emulsion.

**Figure 2:**
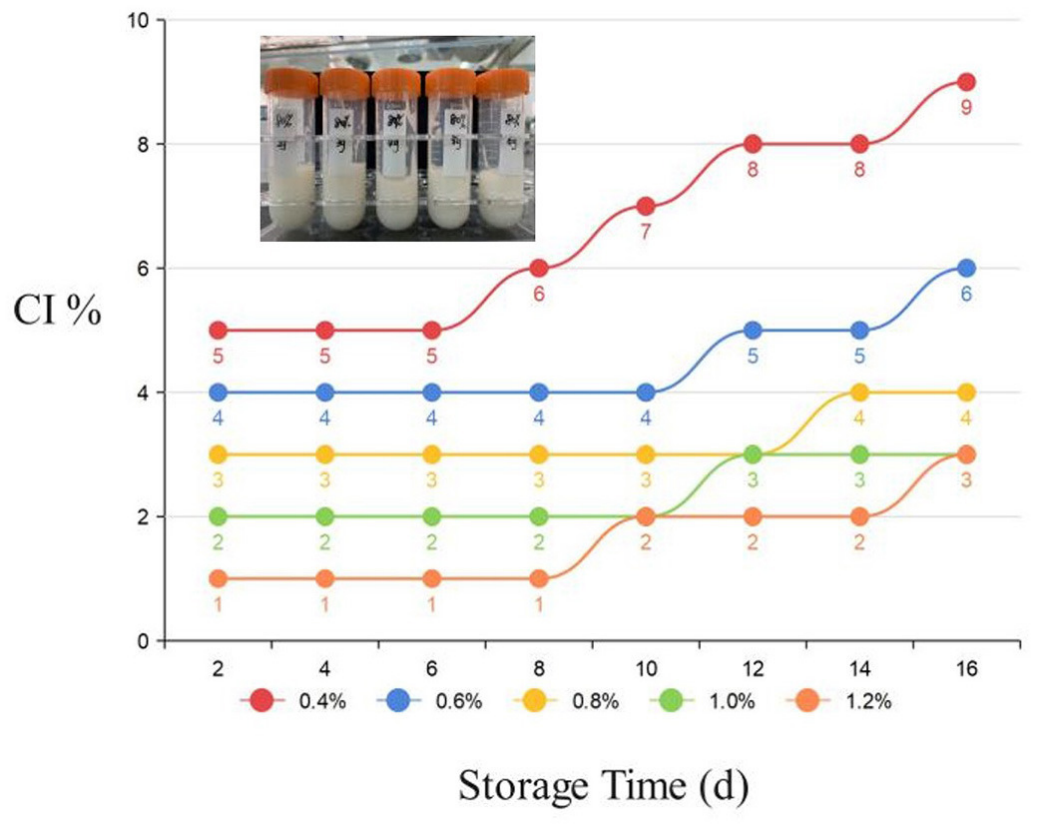
The sample is left standing after high-speed shearing at 12,000r/min for 2 min (from left to right the concentration of A/S-SPIG particles is 0.4, 0.6, 0.8, 1.0, 1.2%).

All mixed samples were left at room temperature for 30 min and no delamination of samples occurred. The apparent viscosities of all samples were very high, which showed typical gel-like characteristics. In Figure 2, during the 20-day storage period, the fat flotation rates of all HIPPEs were maintained at very low level, indicating that the emulsion storage stability was excellent. The fat floating rate further decreased as the concentration of A/S-SPIG particles increased similar to the phenomenon observed by Liu and Tang (12). A possible explanation is that more protein flocs are adsorbed per unit interfacial area (acid induction makes the protein flocculate easily), forming a local flocculation network structure, thereby inhibiting fat floating to a certain extent. At a concentration of 1.2%, HIPPE has the best cohesive property and stability against delamination due to weakened mobility.

### Rheological Test Results

#### Steady Shear Scan Results

**Figure 3:**
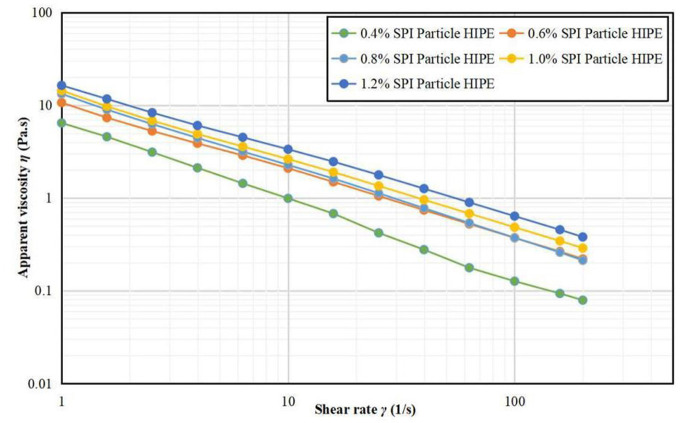
Steady state shear images of A/S-SPIG particle stabilized HIPPE at different particle concentration.

Figure 3 shows that the apparent viscosity of the samples gradually increased with the increase of A/S-SPIG particles, indicating that the emulsion structure was stable with the addition of the appropriate amount of A/S-SPIG particles, which was mainly due to the increase of the concentration of A/S-SPIG particles (24). On the other hand, the apparent viscosity of all emulsions decreased with shear rate increasing, showing a typical shear thinning phenomenon, which indicated that the samples were pseudoplastic fluids (25).

#### Frequency Scan Results

**Figure 4:**
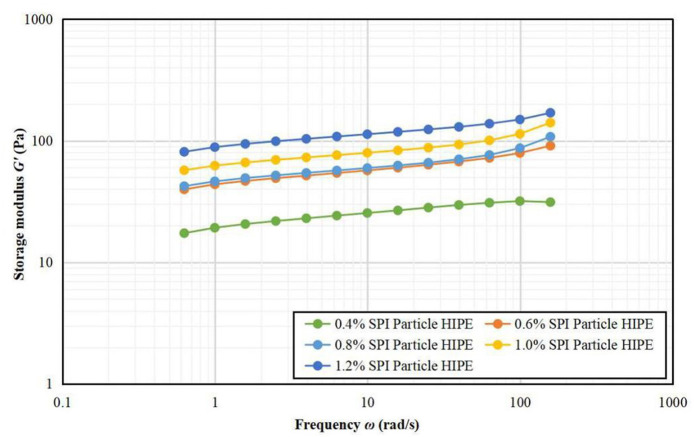
Frequency scanning G' images of A/S-SPIG particle stabilized HIPPE at different particle concentration.

**Figure 5:**
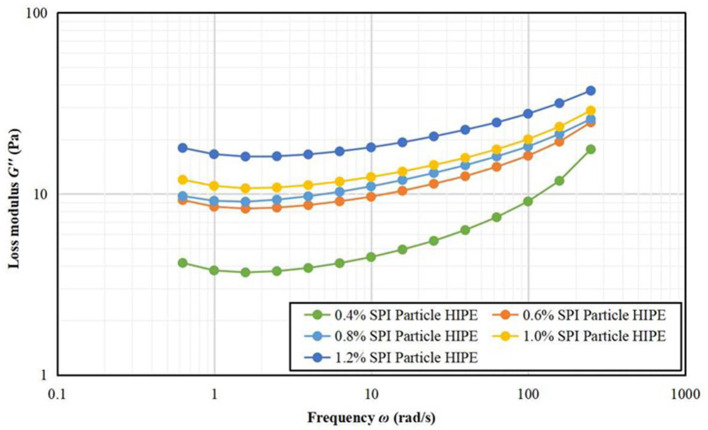
Frequency scanning G” images of A/S-SPIG particle stabilized HIPPE at different particle concentration.

Figures 4, 5 depicts that the storage modulus (*G'*) of all samples is larger than the loss modulus (*G“*) over the entire angular frequency range, indicating that all samples showed solid properties and were elastic. The storage modulus and loss modulus increased steadily with the increase of A/S-SPIG particles, which suggests that both the viscosity and elasticity of the emulsion show positive correlation to frequency. The formation of emulsion network structure is probably because that the high A/S-SPIG particle concentration causes droplet flocculation in a wide range of emulsions (26).

Table 1 lists the results of the power law function fitting for stable HIPPE under different A/S-SPIG particle concentrations. The A/S-SPIG particles were added and the values of k' and k” of the samples increased with the increase of the A/S-SPIG particle concentration. However, the increase showed that with the addition of SPI, the elasticity and viscosity of the HIPPE stabilized by A/S-SPIG particles were enhanced. The value of *n'* did not change, while the value of *n”* decreases significantly, which illustrated that the frequency dependence of the elastic properties of HIPPE remained stable, while the frequency dependence of the viscosity properties decreased with the addition of A/S-SPIG particles. The weakening of viscosity characteristics indicated that the emulsion was mainly chemically cross-linked (26).

**Table 1:** Effects of A/S-SPIG particle stability HIPPE power law function fitting at different particle concentration.

	***G' = K' × ωn*** **'**			***G” = K” × ωn*** **”**		
**A/S-SPIG concentration (%)**	***K'***	***n'***	***R*2**	***K”***	***n”***	***R*2**
0.4	19.758 ± 0.376^a^	0.103 ± 0.006^a^	0.969	2.512 ± 0.097^a^	0.265 ± 0.022^a^	0.958
0.6	42.160 ± 1.023^b^	0.139 ± 0.007^b^	0.974	6.404 ± 0.312^b^	0.192 ± 0.014^b^	0.964
0.8	45.047 ± 0.946^b^	0.130 ± 0.007^b^	0.974	7.210 ± 0.264^b^	0.194 ± 0.011^b^	0.0.980
1.0	60.863 ± 1.183^b^	0.124 ± 0.006^b^	0.975	8.478 ± 0.364^b^	0.177 ± 0.012^b^^,^ ^c^	0.967
1.2	86.478 ± 1.774^c^	0.121 ± 0.006^b^	0.974	12.980 ± 0.481^c^	0.156 ± 0.011^c^	0.967

a,b,c *Values in a column with different superscripts were significantly different (p < 0.05)*.

#### Creep Recovery Test Results

**Figure 6:**
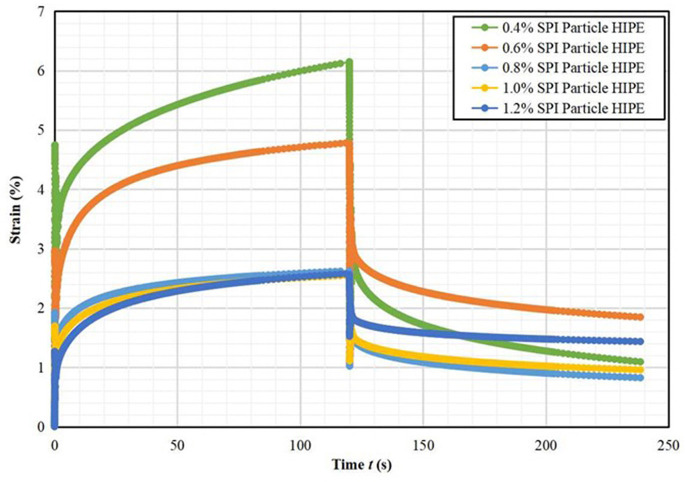
Creep test image of strain of A/S-SPIG particle stabilized HIPPE at different particle concentration.

**Figure 7:**
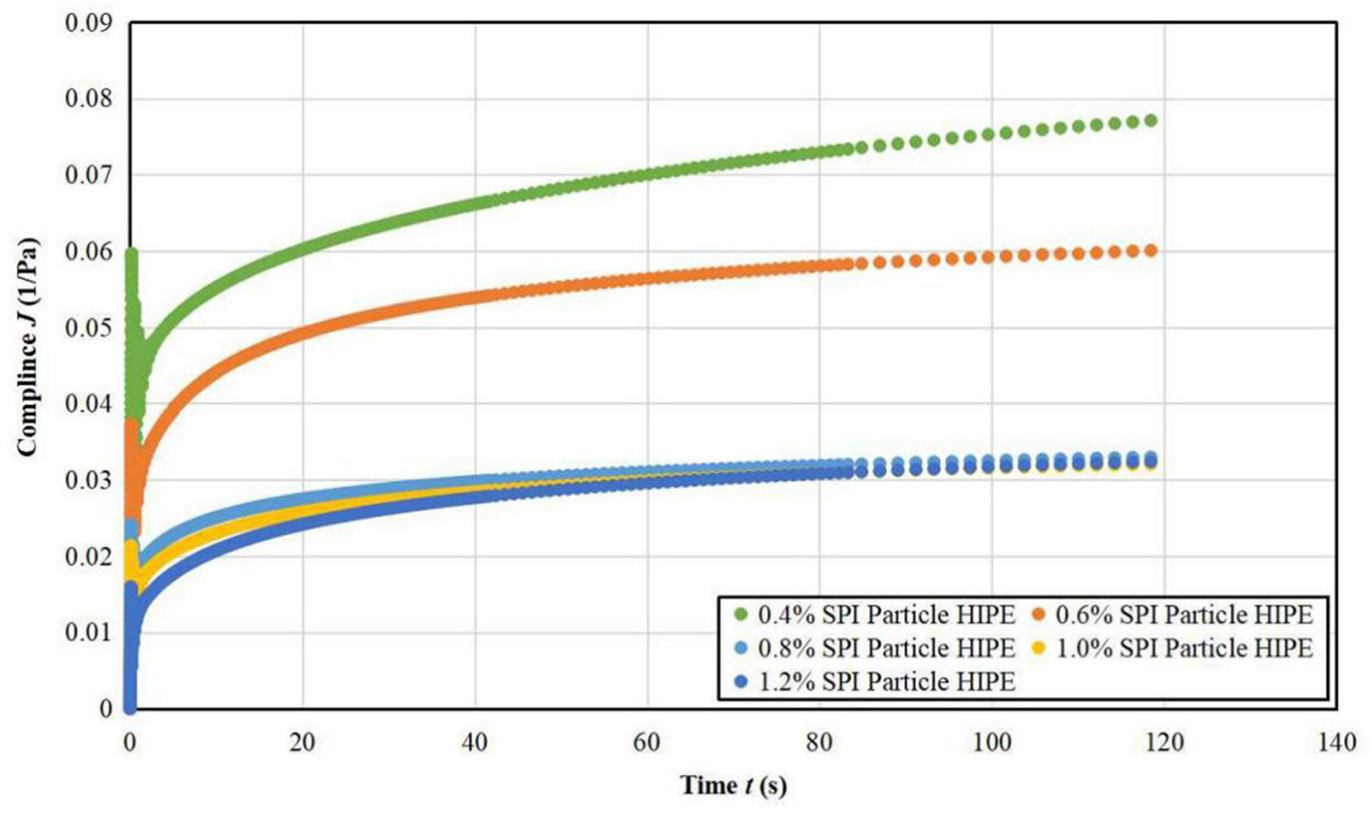
Creep test image of compliance of A/S-SPIG particle stabilized HIPPE at different particle concentration.

It can be seen from Figures 6, 7 that the stable HIPPE under different A/S-SPIG particle concentrations have typical viscoelastic characteristics (27). The maximum creep value (the peak strain at the end of creep) decreased significantly with the increase of A/S-SPIG particle concentration, indicating that the A/S-SPIG particle concentration significantly enhanced the deformation resistance of the system. It showed that with the addition of A/S-SPIG particles, the network structure of HIPPE were more strongly stabilized (28).

The creep compliance of SPI particle stabilized HIPPE with different particle concentrations is fitted by a four-element Maxwell-Voigt model as shown in Table 2. The Maxwell-Voigt model can fit the creep recovery data well (*R*^2^ > 0.999). The G_H_ values of all samples increased with increasing A/S-SPIG particle concentration, indicating that the addition of A/S-SPIG enhanced the instantaneous elastic behavior of A/S-SPIG particles stabilized HIPPE. The hysteresis time τ was almost the same for all simples, meaning that the arrangement of molecules was not affected by the concentration of A/S-SPIG particles, and the orientation of molecular arrangement remained almost unchanged (25).

**Table 2:** Changes in regression parameters of A/S-SPIG particle stable HIPPE creep recovery curve under different SPI particle concentration.

	***G_***H***_***	***G_***V***_***	**τ**	*****η_*n*_*****	***R^**2**^***	*****η_*v*_*****
**A/S-SPIG concentration (%)**	**Pa**	**Pa**	**s**	Pa*s	**%**	Pa*s
0.4	34.242 ± 7.643^a^	51.894 ± 0.239^a^	11.832 ± 0.192^a^	66.459 ± 2.879^a^	0.999	614.009
0.6	51.932 ± 18.564^a^	47.428 ± 0.116^a^	9.386 ± 0.105^a^	90.221 ± 5.603^b^	0.999	445.159
0.8	81.914 ± 32.378^b^	100.005 ± 0.287^b^	9.547 ± 0.131^a^	156.566 ± 11.828^c^	0.999	954.747
1.0	89.064 ± 18.194^b^	92.65 ± 0.270^b^	10.51 ± 0.124^a^	191.508 ± 8.412^d^	0.999	973.751
1.2	173.315 ± 8.799^c^	73.702 ± 0.218^c^	11.399 ± 0.125^a^	141.659 ± 5.878^c^	1.000	840.129

a,b,c,d *Values in a column with different superscripts were significantly different (p < 0.05)*.

The effect of different A/S-SPIG particle concentration on the creep recovery rate of SPI particle stable HIPPE is presented in Table 3. The recovery of the emulsion was 82.22% at the A/S-SPIG particle concentration of 0.4% and decreased to 44.46% with the increase of the A/S-SPIG particle concentration. It illustrated that the increase of A/S-SPIG particle concentration made HIPPE easy to be destroyed.

**Table 3:** The effect of different SPI particle concentration on the creep recovery rate of SPI particle stable HIPPE.

**A/S-SPIG concentration (%)**	**Recovery rate (%)**
0.4	82.222 ± 5.326^a^
0.6	61.386 ± 4.287^b^
0.8	68.590 ± 4.112^b^
1.0	62.473 ± 6.826^b^
1.2	44.463 ± 2.965^c^

a,b,c *Values in a column with different superscripts were significantly different (p < 0.05)*.

### Particle Size Test Results of HIPPE Droplets

**Figure 8:**
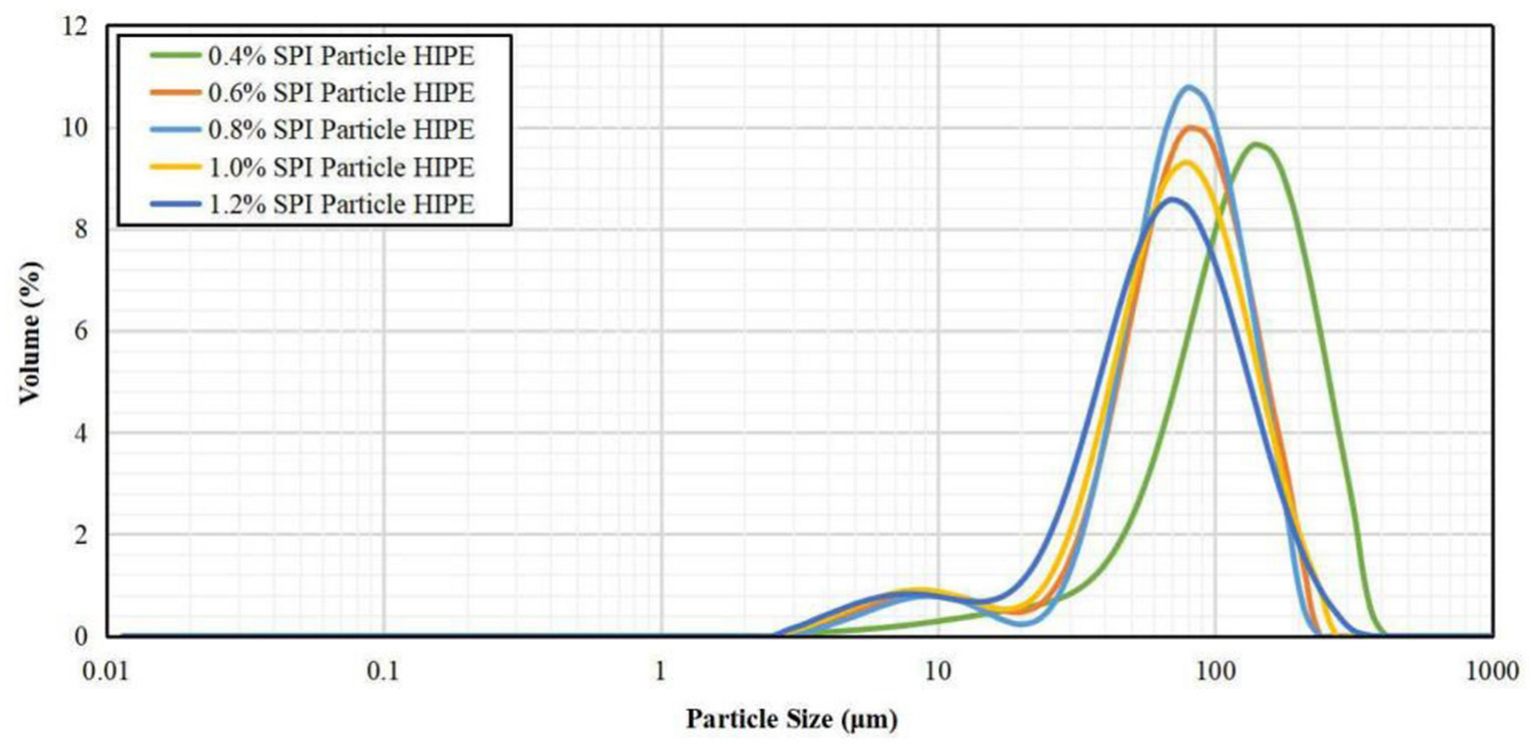
Particle size distribution of SPI stable HIPPE with different particle concentration.

As shown in Figure 8, the lower peaks of the particle size curves were very low and similar, indicating that all the free SPI particles in HIPPE did not produce significant aggregation. The higher peak of particle size curve represented the droplet diameter of HIPPE, and the average particle sizes of HIPPE droplets with a concentration of 0.2% SPI particles were about 130 microns. As the concentration of SPI particles increases, the average particle sizes of the HIPPE droplets gradually decreased, showing a downward trend, which also reflected that increasing the particle content could stabilize a larger interfacial area. It indicated that increasing the concentration of SPI particles can effectively improve the emulsification effect of HIPPE (29).

### Freeze-Thaw Stability Test Results

**Figure 9:**
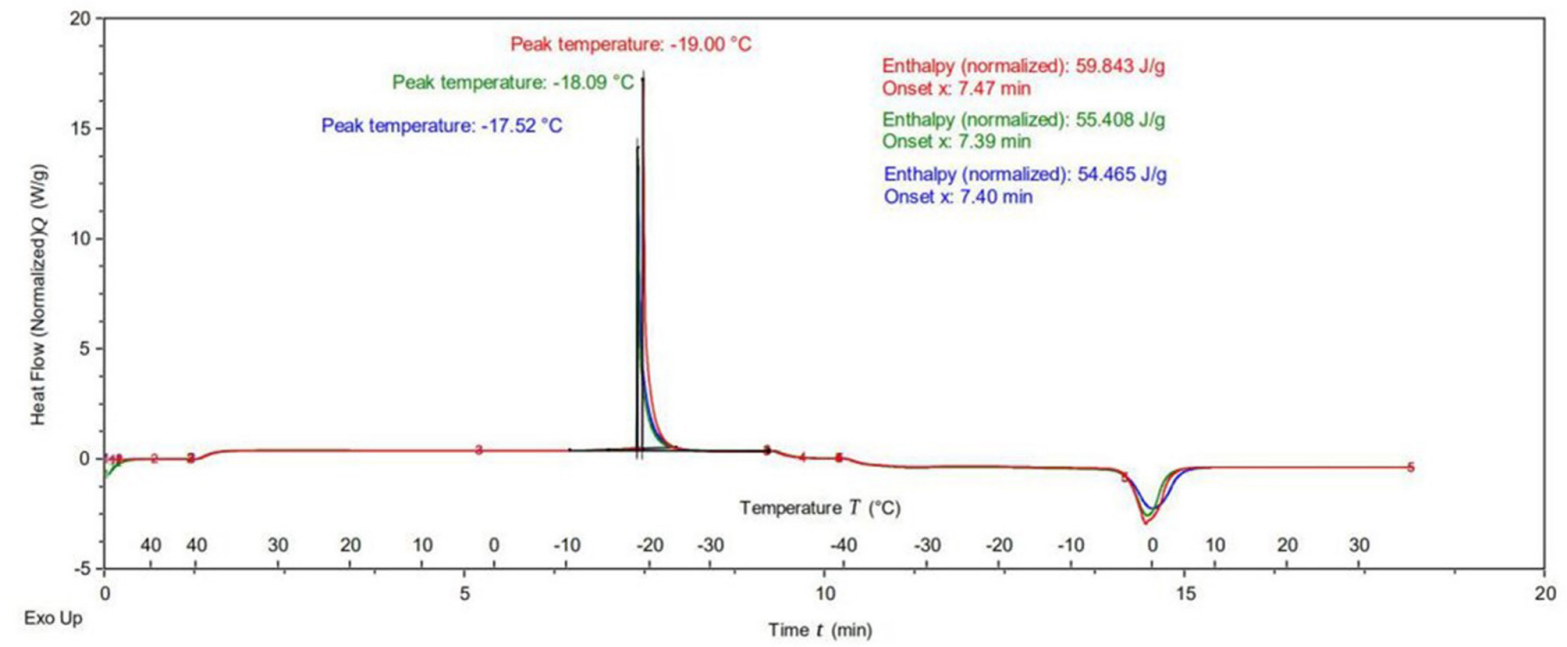
The effect of different A/S-SPIG particle concentration on the freeze-thaw stability of SPI particle stable HIPPE.

Figure 9 depicts that the exothermic peak of the HIPPE in cooling and freezing process gradually increased from −19.0 to −17.5°C, the endothermic peak in heating and melting process was about −1.0°C. This is similar to the results of freeze-thaw stability tests of modified starch stabilized emulsions (30). The total enthalpy value of the HIPPE decreased from 59.84 to 54.46 J/g, which improved the thermal stability of the HIPPE system. The freezing point of an emulsion is an important factor affecting its freeze-thaw stability. The heat release and endothermic peak temperature indicated the crystallization and melting temperature of the oil-water two-phase in the emulsion system. When the concentration of SPI particles increased from 0.8 to 1.2%, the temperature of the exothermic peak increased by 2 degrees, indicating that the crystallization temperature of the emulsion increased. It proved that the high concentration of SPI particles increased the crystallization temperature of the stabilized HIPPE.

### Microstructure of HIPPE

**Figure 10:**
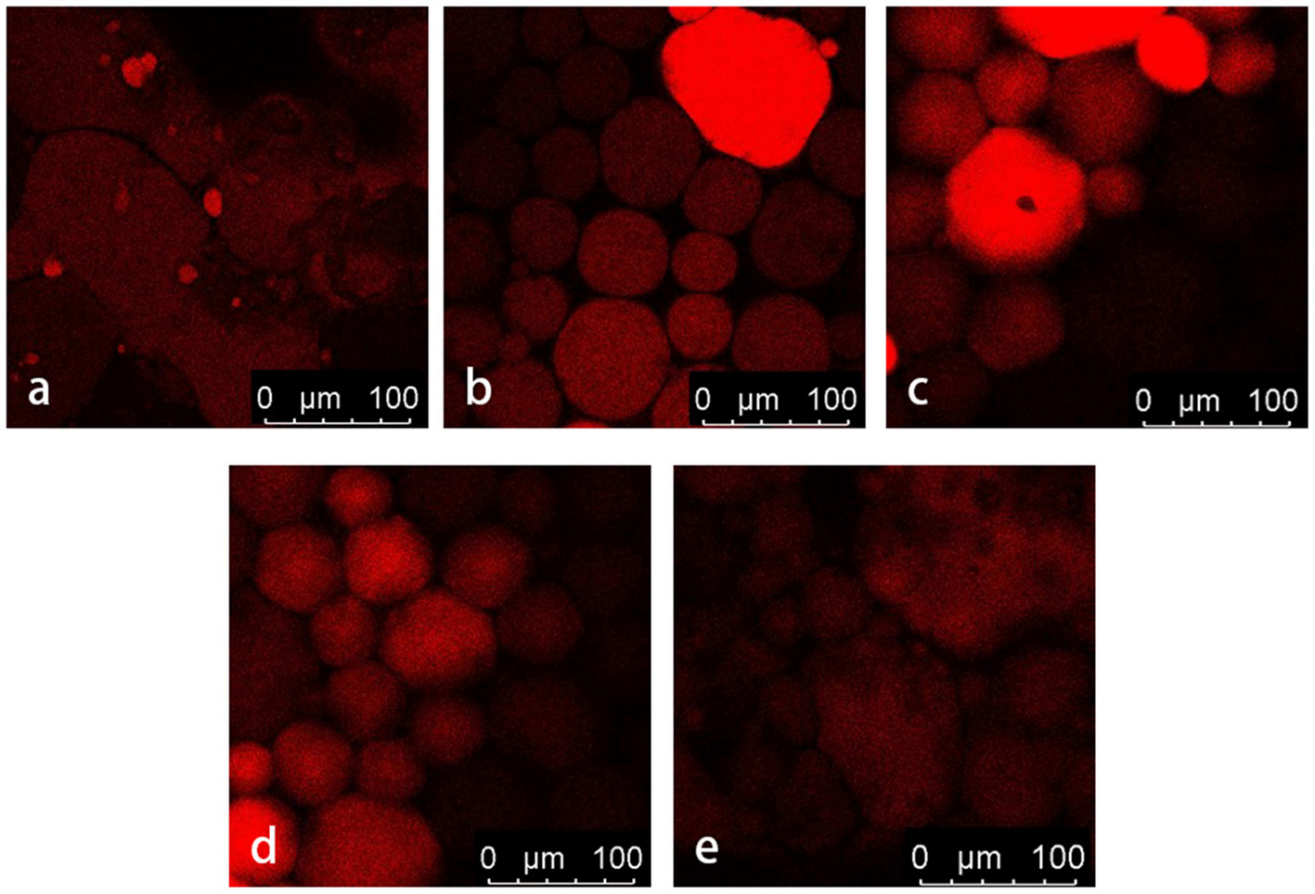
The effect of different SPI particle concentration on the microstructure of stable HIPPE [**(a)** 0.4% SPI particle concentration, **(b)** 0.6%, **(c)** 0.8%, **(d)** 1.0%, **(e)** 1.2%].

Figure 10 shows CLSM images of A/S-SPIG particle stabilized HIPPE at different particle concentrations. The protein enrichment region of Nile Red staining is red. The HIPPE containing 0.2% A/S-SPIG particles were mainly composed of sheet-like networks. Large droplets were dispersed in the system of sheet-like form, which were not effectively encapsulated. As the concentration of A/S-SPIG particles increased to 1.0%, the droplet size of the HIPPE gradually decreased and changed to the microstructure of concentrated droplets. The formed oil droplets were tightly wrapped by A/S-SPIG particles. As the concentration of A/S-SPIG particles increased to 1.2%, an oil-water emulsion was formed, which indicated that the amount of protein exceeded the requirement of forming HIPPE. In addition, the A/S-SPIG particles were mainly adsorbed on the surface of oil droplets, and only a few A/S-SPIG particles were free in water. This meant that A/S-SPIG particles can be well-adsorbed, which had obvious potential as a Pickering stabilizer.

## Conclusion

In this paper, from the perspectives of rheology, thermodynamics, and microstructure, the potential of A/S-SPIG particle as efficient HIPPE stabilizer was studied. The result showed that gradually increasing the concentration of A/S-SPIG particles in the emulsion system could strengthen the elasticity and viscosity properties of HIPPE. The high concentration of A/S-SPIG particles led to the formation of a strong network structure, while the toughness of the emulsion was reduced. The frequency dependence of the system's viscosity characteristics decreased with the increase of the A/S-SPIG particle concentration. The increase of A/S-SPIG particle concentration effectively improved the emulsification effect of HIPPE, and the average particle sizes of emulsified droplets gradually decreased. The high concentration of A/S-SPIG particles could improve the freeze-thaw stability of HIPPE to a certain extent. The good adsorption of A/S-SPIG particles could improve the stability of HIPPE. In conclusion, A/S-SPIG particles have great potential as Pickering stabilizers.

## Data Availability Statement

The original contributions presented in the study are included in the article/supplementary material, further inquiries can be directed to the corresponding authors.

## Author Contributions

C-hB: conceptualization, methodology, and funding acquisition. S-yC: writing—review and editing. TZ and J-yZ: review and editing. X-yW: investigation, software, and writing. Z-gH: methodology, supervision, and writing. FG: methodology. All authors contributed to the article and approved the submitted version.

## Funding

This research project was supported by the National Key Research and Development Program of China (2018YFD0400804), 2021 Postgraduate Research Ability Improvement Program BTBU, Beijing Natural Science Foundation (6184036), and Beijing Excellent Talent Training Project (2017000020124G100).

## Conflict of Interest

The authors declare that the research was conducted in the absence of any commercial or financial relationships that could be construed as a potential conflict of interest.

## Publisher's Note

All claims expressed in this article are solely those of the authors and do not necessarily represent those of their affiliated organizations, or those of the publisher, the editors and the reviewers. Any product that may be evaluated in this article, or claim that may be made by its manufacturer, is not guaranteed or endorsed by the publisher.
